# Status and innovation needed to address health disparities in opioid use disorders among hispanic pregnant individuals

**DOI:** 10.3389/fgwh.2025.1575164

**Published:** 2025-05-29

**Authors:** Karen G. Martinez-Gonzalez, Darlene I. Santiago

**Affiliations:** ^1^ Department of Psychiatry, University of Puerto Rico, Medical Sciences Campus, San Juan, Puerto Rico; ^2^ School of Pharmacy, University of Puerto Rico, Medical Sciences Campus, San Juan, Puerto Rico

**Keywords:** perinatal opioid use disorder, perinatal mental health, hispanic, collaborative care, opioid use disorder

## Abstract

Although opioid use disorder (OUD) in pregnancy has increased significantly in the last years, there are still significant gaps in scientific data and in access to evidence-based treatments. OUD in pregnancy is associated with negative health outcomes in the pregnant person, the fetus, and the newborn. To prevent these consequences, it is imperative to identify OUD and provide treatment as soon as possible in the pregnancy. Effective treatments, such as medication for opioid use disorder (MOUD), are safe in pregnancy but not routinely prescribed. For Hispanic pregnant people, these evidence-based treatments are less likely to be prescribed, are less consistently used and are less likely to be continued during the first year postpartum. Increasing access to high quality evidence-based treatments for OUD in Hispanic pregnant people is a public health emergency. This article will offer an overview of the known health disparities of treating perinatal OUD in Hispanics and propose strategies to address these disparities.

## Introduction

### Opioid use and pregnancy

Opioid use during pregnancy has significantly increased in the last decade (131% from 2010 to 2017), with drug overdose mortality rising by approximately 81% for this population (1). Individuals that, at the time of delivery, had opioid use have been found to be 4.6 times more likely to die during hospitalization and 3.5 times more likely to have postnatal birth-related complications (cardiac arrest, premature birth, blood transfusion, stillbirth, cesarean section, and preeclampsia) (1). Opioid use is one significant cause for the high morbidity and mortality rates in pregnancy in the United States (US) (2, 3). Survey data from the Centers for Disease Control (CDC) show that in 2019, 7% of women reported prescription opioid use during pregnancy. Of these, approximately 21% reported misuse, 27% wanted to stop or reduce consumption, and almost 32% reported not receiving provider counseling on the effects of opioid misuse (4).

### Opioid use disorder in pregnancy

Compulsive opioid use despite harmful consequences is known as opioid use disorder (OUD), and it presents unique and severe risks for the pregnant individual (5). Undiagnosed and untreated opioid use increases the risk of maternal mortality (6, 7) by overdose, cardiac arrest, placental abruption, increased length of hospitalization, transfusion, and increased rates of cesarean delivery (8, 9). In addition, intrauterine opioid exposure increases the risk to the fetus for developmental defects, preterm labor, neonatal abstinence syndrome (NAS), long-term- developmental outcomes for the newborn (10) and post-neonatal infant mortality (11). Increases in the rates of infants in foster care in the US have also been attributed to OUD (2). To prevent these consequences, it is imperative to identify OUD during pregnancy and provide treatment as soon as possible (5, 12). In addition, the perinatal period has been shown to be a critical period when people are more open to start OUD treatment as they frequently interact with healthcare services (13).

Effective pharmacological treatments such as medication for opioid use disorder (MOUD) with either buprenorphine or methadone are safe and recommended to use during pregnancy (1). Buprenorphine is preferred over methadone due to its safety, efficacy, and the ease with which it can be obtained, such as filling a monthly prescription at a pharmacy. Unlike methadone, which requires the individual (or pregnant individual) to attend a specialized clinic daily to obtain the medication in person. Although these medications are safe, available, and proven to increase the outcomes of the pregnant individual and the newborn, many do not receive pharmacotherapy for OUD during pregnancy. For pregnant people from minority groups, these MOUD treatments are less likely to be prescribed, are less consistently used, and are less likely to be continued during the first year postpartum (1). Recent studies demonstrate differences by race and ethnicity of receipt of MOUD pharmacotherapies, with Hispanic and Black pregnant and postpartum individuals having lower buprenorphine or methadone use rates (3, 14–16).

Pharmacological treatments effectively reduce the adverse effects of opioid use. However, harm reduction strategies can be an effective alternative even during pregnancy (17). Perinatal harm reduction promotes beneficial practices, thus eliminating harmful activities and risk factors that can impact a parent's ability to care for their children and unborn. Traditionally harm reduction has been associated with distribution of naloxone kits, syringe services programs, medications for opioid use, medication lock boxes and/or blood borne infection testing. On the other hand, perinatal harm reduction strategies can involve setting goals for abstinence, decreased use, and safer use, and can expand to addressing other social risk factors such as getting a home or a job and enrolling in health care and perinatal care if necessary (18). The Academy of Perinatal Harm Reduction has created a toolkit on pregnancy and substance use, including alcohol, benzodiazepines, cannabis, opioids, stimulants, and tobacco. It provides guidance on navigating the care and legal systems, prenatal care, labor, childbirth, and postpartum care (18). A Spanish version is also available. It is important that perinatal healthcare workers provide harm-reduction services within their therapeutic relationship to promote safety, respect, and patient autonomy (17).

### Challenges for OUD treatment and retention during pregnancy

Accessing and maintaining treatment for OUD during pregnancy presents numerous challenges. Pregnant individuals with opioid use often face stigma and discrimination within the healthcare system and from society, which can discourage and prevent them from receiving much-needed healthcare services (screening, treatment, and medication). Fear, guilt, and shame can exacerbate the lack of access to treatment for pregnant individuals, specifically from minority groups and communities of color (19), who already experience higher rates of peripartum opioid use (20). Involvement of child protection services is a common variable among pregnant individuals with opioid use (21); experiences of these individuals illustrate the fear of being separated from their families or losing custody of their children while seeking help or treatment for opioid use, conveying a sense of bewilderment at the idea of seeking treatment and being penalized simultaneously. Unmet social needs such as food and house insecurity are another challenge preventing pregnant individuals from receiving care for opioid use (22), specifically in rural communities. Co-occurring mental health challenges, fear, and isolation are additional barriers faced by these individuals (22); these can further complicate access and retention in treatment.

Maintaining access and long-term engagement in treatment for pregnant individuals requires overcoming these challenges by developing groundbreaking and specialized interventions that can transform current professional guidelines into evidence-based, efficacious interventions. The American College of Obstetricians and Gynecologists (23) recommends routine universal screening for substance use starting on the first prenatal visit. Screening tools aid in timely identification and referral to treatment, lessening stereotyping and stigma of toxicology and drug testing (22). Pharmacotherapy with MOUD and behavioral therapy, being the standard of treatment for OUD, can successfully address the unique challenges faced by pregnant individuals who use opioids, but they need to be referred to these treatments.

### Opioid use disorder in hispanics

Pregnant individuals who use opioids deserve compassionate and efficacious interventions that reduce its negative consequences and life-threatening effects, regardless of race or ethnicity. When analyzed by race and ethnicity, most of the literature reports a higher severity of OUD for White individuals in the perinatal period. However, evidence exists that Hispanic pregnant individuals are less frequently prescribed medication for opioid use, and even when prescribed, are less likely to consistently use medication for treatment during pregnancy, compared to White non-Hispanic pregnant individuals with OUD (24). The lack of visibility of specific medical and clinical outcomes for Hispanic pregnant individuals in the literature, alongside reported lower rates of medication usage, positions Hispanic pregnant individuals at a heightened risk and greater severity for adverse and life-threatening outcomes. It is imperative that innovative interventions are evaluated that address access to timely diagnosis and treatment for Hispanic pregnant individuals with opioid use, reducing the current critical and life-threatening scenario for these individuals. OUD in Hispanic pregnancy is a public health emergency; its intersection creates a critical window of opportunity for transformative interventions that can significantly enhance the outcomes of the pregnant individuals and neonates.

### Health disparities in hispanics

Hispanics make up more than 19% of the population of the US, making them the largest minoritized and ethnic group (25). People who identify as Hispanic, Latino, or Spanish share a common heritage but represent a variety of country of origins as well as different social determinants of health. Although the scarce literature available on perinatal OUD aggregates data as Hispanics, other health conditions have identified the need to evaluate the health status and need of each Hispanic group. In addition, when looking at health disparities by race or ethnicity, it has been commonly stated that they could serve as a proxy for other variables such as social determinant of health or structural factors for health (26). Knowing the limitations of the data, significant health disparities have been described in Hispanics as aggregates, including increased rates of asthma, chronic obstructive pulmonary disease, HIV/AIDS, suicide and liver disease (27). Although some improvements have been made in disparities regarding physical health, access to mental health services seems to be getting worse for Hispanics (27). In addition, structural, economic, cultural/social norms and mental factors, related to the devaluation and stigma of psychological suffering, as compared with the physical suffering, contribute to the illness manifestation and therefore its treatment (28). There are also cultural factors in the way distress is expressed and managed. Studies have suggested that Hispanics present a sensibility to anxiety or “fear to fear”, which predisposes to *ataque de nervios* (nerve attacks). Rubio et al. first described this syndrome as an intense emotional outburst among Puerto Rican army recruits. It was then conceptualized as a sociocultural expression of distress called “*Puerto Rican syndrome*”. The presence of *ataque de nervios* in clinical settings has been associated with greater suicidal ideation, disability and outpatient psychiatric utilization, even after adjusting for the contribution of comorbid psychiatric diagnosis, trauma exposure and other covariates. These studies also suggest that *ataque de nervios* seem to identify those who suffer from a combination of social disadvantage, psychiatric disorders, and poor perceived health. These studies on *ataque de nervios* also highlight that Hispanics express distress with physical symptoms (29). The literature on health disparities and cultural expression of disease among Hispanics propose that diseases such as OUD should be examined through a lens that takes these factors into consideration.

### Health disparities in perinatal OUD among hispanics: Puerto Rico as a case study

The social determinants of health that affect women in Puerto Rico (PR) place them at a higher risk of perinatal mental health disorders. In 2019, the poverty rate in PR (43.5%) was higher than the poorest state in the US (Mississippi,19.6%). Puerto Rican women have a higher probability of being the head of a household under poverty level (65.6%). With regards to environmental stressors, in the last 5 years Puerto Rican women have lived through a Zika epidemic, two major hurricanes in 2017 and, right before the COVID 19 pandemic, PR suffered from a sequence of earthquakes that left many with home insecurity. Puerto Rican mothers are also disproportionately exposed to other risk factors for perinatal mental health disorders. For example, Puerto Rican mothers had higher rates of adverse obstetric outcomes with the cesarean section birth rate at 47% and premature birth rate at 11.8%. The maternal mortality rate in PR for 2016 was 31.9 deaths for each 1,000 live births. The rates of domestic violence in PR are also staggering with 6,725 cases reported in 2019 (30–33).

Among Hispanics in the US, Puerto Ricans are born with US citizenship status and have been found to experience the worst health outcomes, including being more likely to die of drug overdoses (26). For this reason, OUD in PR is also a public health emergency. In 2021, a law (Ley Num. 35 de 27 de Agosto de 2021; Law Num. 35 of August 27, 2021) was created to prevent opioid overdose deaths by removing obstacles that are known to prevent overdose reversion with Naloxone and individuals seeking treatment after illicit opioid use (34). Several local news outlets have reported the severity of the opioid crisis, reporting on unique overdosed deaths, opioid use in older adults (35), overdose deaths within incarcerated individuals (36), how shipping of Naloxone for opioid reversal during environmental emergencies such as hurricanes was needed due to increased overdose deaths (37), how Covid-19 only worsen the opioid crisis in the island (38), and lastly but not less important increased opioid use during pregnancy (39). The latter reported a quadruple increase in pregnant women with OUD over a fifteen-year period ending in 2014 per data from the CDC, a 333% increase in the local prevalence of pregnant women in hospitals on the island (39). Trustworthy and statistically comprehensive assessments of the mortality and severity of the opioid crisis on the island are limited. Despite the government's recognition of this issue, a state of national opioid emergency was declared for the island, leading to the creation of a Task Force in 2017 (*task force para la prevención de sobredosis por opiáceos* or task force for the prevention and overdose of opioids in English). That Task Force aimed to address the island's opioid overdose deaths by uniting local and federal governments and community-based organizations. Although the perinatal population has been identified by the PR Department of Health as an area of special need in these opioid crisis interventions, there is a lack of scientific data that can guide these efforts.

In individual interviews with members of the perinatal workforce, we have identified several areas of opportunity on how to start addressing OUD in the perinatal period in PR. A community midwifes/ “*partera*” was first skeptical about how many people in the perinatal period we would find with substance use disorders. When we presented her with the available data on substance use in pregnancy in our community, she recounted an experience early in her career when she asked a patient about substance use who responded with anger and left her service. She told us she was hesitant about asking about substance use after that, which is why she might have not noticed this is a problem within our perinatal community. An obstetrics and gynecology specialists talked about her frustration of knowing that patients had an active substance use disorder but having nothing to offer them. She worries about voicing her concerns to the clinic's social workers as she fears they will contact the Department of Family Services instead of referring for specialized substance use services. She narrated countless examples of how substance use disorders are seen in high-risk obstetric clinics from patients who disappear from their rooms and return intoxicated to the reluctance of some to receive pain management during birth.

An important aspect that both perinatal workforce members highlighted was the lack of adequate pain management during the birth process. Most healthcare plans in PR do not cover epidural pain management and thus patients have only intravenous or intramuscular opioids as pain management treatments during birth. From these interviews with members of the perinatal workforce in PR, it was evident that they lacked the training and referral sources to deal with substance use disorders in this population. It was clear that our perinatal workforce needed tools to start screening for substance use disorders as a first step to address this opioid emergency. When we then asked our collaborators in the addiction field, they asserted that the substance use disorders clinics in PR are open to pregnant people but that they cannot recall ever receiving a referral for treatment for a pregnant person. We have two different healthcare environments- the perinatal workforce and the addiction workforce- that are not working together to address the opioid use crisis in pregnancy, at least within our community. Although there are several strategies to start addressing perinatal OUD in PR, including policy changes in healthcare coverage and increasing access to specialized interdisciplinary perinatal health services, screening and identification of those at risk or with OUD is an essential first step.

### Steps needed for creating a collaborative care environment that works for addressing health disparities in perinatal OUD among hispanics

1) Increase the data on prevalence and risk factors for OUD in Hispanics including disaggregated data among the specific Hispanic groups. Having more information on specific needs will support the development of screening, assessment and treatment programs that can be effective for Hispanics. In PR, there are several initiatives trying to identify data sources such as the Center for Disease Control and Prevention (CDC) Pregnancy Risk Assessment Monitoring System (PRAMS) that could include data on opioid use. In addition, data is being gathered and disseminated about opioid use such through a publicly available dashboard (https://datosopioides.pr.gov/) but specific data related to perinatal OUD is still not available. It is important to identify data sources with other Hispanic groups in the US, including Puerto Ricans on the mainland.
2) Improve screening of OUD in perinatal health settings. Screening, Brief Intervention, and Referral to Treatment (SBIRT) has been defined as one of the evidence-based early interventions for substance use disorders by the Substance Abuse and Mental Health Administration (SAMHSA) (40). It was originally designed to provide early intervention for alcohol use disorders in non-substance use services. The evidence has evolved to prove its effectiveness as a brief, universal screening method for all types of substance use disorders. It is a billable procedure that can be provided by primary care physicians, specialty physicians, dentists, chiropractors, social workers, nurses, nurse practitioners, and/or physician assistants. It has been adapted to be used with adolescents, adults, and pregnant populations. In pregnancy, it has been used for the prevention of fetal alcohol disorder and more recently for early identification and management of opioid use disorder (41–43). Although SBIRT has been shown to be effective in diverse clinical populations (44), to our knowledge, there are no current efforts of SBIRT integrated into perinatal care in PR at this time.
  The first step to identification and referral to treatment is screening. A specialized screening instrument for pregnant women is the Institute for Health and Recovery's Integrated 5 P'S Screening Tool. This tool is based on the Parents, Partner, Past and Pregnancy (4 P's) screening tool (45, 46). The tool asks about parents, friends and partner alcohol or other drug use and feels unsafe in the relationship with current partner before asking about past and present alcohol and substance use. The screening also includes a question asking for worry, anxiety, depression, or sadness. The questionnaire then guides the interviewer on when and how to intervene depending on which questions were answered positively (yes) in the screening tool. The 5 P's screening tool has been shown to be the most sensitive screening tool for substance use disorders in pregnant populations (36). It would be important to culturally validate this questionnaire in Spanish for different Hispanic groups, including Puerto Ricans. At this time, there is no known OUD screening questionnaire in Spanish that has been validated in PR.
3) Train perinatal health workforce in nonpharmacological interventions that can be used in non-mental health settings such as motivational interviewing skills. Although motivational interviewing is commonly identified as an effective intervention for perinatal OUD (47), it is not commonly used in perinatal services in Puerto Rico. To address these gaps in OUD screening within perinatal services, culturally adapted brief interventions based on motivational interviewing techniques can be helpful to identify obstacles and promote factors toward seeking treatment for substance use disorders. The evidence-based techniques that can be easily taught to non-mental health professionals include strategies to create collaborative, goal-oriented conversations with patients to promote change (48). Some specific skills that the perinatal workforce can learn include the use of open-ended questions, affirmations, reflections, and summaries as techniques to promote the acceptance of treatment engagement for those patients identified as elevated risk of substance use disorder in the screening.
4) Connect perinatal health services with OUD treatment services. Another important aspect is how to connect identified substance use disorder treatment centers with perinatal services. Offering information on how to contact and refer patients to a variety of treatment centers that include state administered specialized clinics, federally health qualified centers and private clinics is essential. These specialized OUD services provide treatment both in person and telehealth services. In PR, there are several treatment options that are free of cost as well as covered by Medicaid and other private health care plans. The rationale of having a variety of services is to provide patients with options that can address treatment barriers such as lack of transportation, stigma, and costs of services. In addition to specialized substance use disorder clinics, the referral guide should include perinatal psychiatry and psychology services, community-based organizations focused on pregnancy and infancy, services for interpersonal violence, and government agencies that can assist with housing, employment, and health care services. In this manner, we address the treatment aspects of OUD but also the social determinants of health that can be contributing to the disorder.
5) Collaborative Care: In our clinical services in PR, there is currently no collaboration between the perinatal workforce and the substance use disorders workforce. It is important that each workforce area understands how each other works. Education on the roles and characteristics of each member of the perinatal team, the challenges that each member of the perinatal care team encounters and how to promote effective interprofessional communication is needed for OUD specialized professionals. In the same way, perinatal professionals could benefit from education on the roles and challenges of the OUD workforce. It is also important to identify strategies to promote interprofessional care such as being non-judgmental, promoting trust, and shared decision making.
6) Harm reduction: Training on traditional harm reduction strategies and targeted perinatal harm reduction can be another key area to address perinatal OUD. Training on the Academy of Perinatal Harm Reduction toolkit on pregnancy and substance use in Spanish can be provided to both perinatal and OUD professionals. This training provides guidance on navigating the care and legal systems, prenatal care, labor, childbirth, and postpartum care. Through harm-reduction training, health professionals can learn important skills such as the need to promote safety, respect, and patient autonomy within the therapeutic relationship.

## Conclusions

There is a significant gap in terms of data on Hispanic perinatal OUD prevalence and treatment. There is even less data on specific Hispanic groups. For this reason, not only is there a need for more research but there is also an urgent need for a compassionate and interdisciplinary OUD/prenatal care model that improves access and retention in treatment. We propose that a few components of a model of collaborative care between perinatal health professionals and OUD specialists can be a simple, cost-effective manner of addressing the public health emergency of perinatal OUD, especially for Hispanic populations. The components that can be easily addressed would be establishing a Screening, Brief Intervention, and Referral to Treatment (SBIRT) with a collaborative care and harm reduction framework.

## Data Availability

The original contributions presented in the study are included in the article/Supplementary Material, further inquiries can be directed to the corresponding author.
